# Covalent functionalization of reduced graphene oxide with porphyrin by means of diazonium chemistry for nonlinear optical performance

**DOI:** 10.1038/srep23325

**Published:** 2016-03-24

**Authors:** Aijian Wang, Wang Yu, Zhipeng Huang, Feng Zhou, Jingbao Song, Yinglin Song, Lingliang Long, Marie P. Cifuentes, Mark G. Humphrey, Long Zhang, Jianda Shao, Chi Zhang

**Affiliations:** 1 China-Australia Joint Research Center for Functional Molecular Materials, School of Chemical Science and Engineering, Tongji University, Shanghai 200092, P.R. China; 2 China-Australia Joint Research Center for Functional Molecular Materials, Scientific Research Academy, Jiangsu University, Zhenjiang 212013, P.R. China; 3 School of Physical Science and Technology, Soochow University, Suzhou, 215006, P.R. China; 4 Research School of Chemistry, Australian National University, Canberra ACT 2601, Australia; 5 Key Laboratory of Materials for High-Power Laser, Shanghai Institute of Optics and Fine Mechanics, Chinese Academy of Sciences, Shanghai 201800, P.R. China

## Abstract

Reduced graphene oxide (RGO)-porphyrin (TPP) nanohybrids (RGO-TPP **1** and RGO-TPP **2**) were prepared by two synthetic routes that involve functionalization of the RGO using diazonium salts. The microscopic structures, morphology, photophysical properties and nonlinear optical performance of the resultant RGO-TPP nanohybrids were investigated. The covalent bonding of the porphyrin-functionalized-RGO nanohybrid materials was confirmed by Fourier transform infrared spectroscopy, Raman spectroscopy, X-ray photoelectron spectroscopy, transmission electron microscopy, and thermogravimetric analysis. Attachment of the porphyrin units to the surface of the RGO by diazotization significantly improves the solubility and ease of processing of these RGO-based nanohybrid materials. Ultraviolet/visible absorption and steady-state fluorescence studies indicate considerable π-π interactions and effective photo-induced electron and/or energy transfer between the porphyrin moieties and the extended π-system of RGO. The nonlinear optical properties of RGO-TPP **1** and RGO-TPP **2** were investigated by open-aperture Z-scan measurements at 532 nm with both 4 ns and 21 ps laser pulses, the results showing that the chemical nanohybrids exhibit improved nonlinear optical properties compared to those of the benchmark material C_60_, and the constituent RGO or porphyrins.

Graphene comprises two-dimensional planar sheets of sp^2^-bound carbon atoms arranged in a hexagonal crystal lattice; it possesses remarkable electronic, optical, and mechanical properties^1^,^2^,^3^, such as high charge-carrier mobility^4^, mechanical stiffness^5^, and excellent nonlinear optical response^6^. However, pristine graphene is unsuitable for further processing because the strong π-π and Van der Waals interactions in bulk graphene result in a pronounced tendency to agglomerate; this can have a significant and detrimental effect on material properties^7^. As a result, considerable effort has been expended in improving the dispersion and compatibility of graphene (including the related graphene oxide, GO and reduced graphene oxide, RGO) by derivatization using noncovalent interactions and, more importantly, covalent-coupling, in order to obtain new graphene derivatives for specific applications^8^,^9^,^10^,^11^. Absorption of functional moieties on the surface can tune graphene’s electronic properties, but this approach is not controllable and does not afford stable adducts^12^. A covalent chemistry strategy is more promising due to the strong bonds forming stable adducts^13^.

The modification of graphene via diazonium functionalization is emerging as a versatile approach for tailoring the chemical and electronic properties of graphene, and has been shown to dope the graphene and open a band gap^14^. Photophysical and photoelectric investigations of the covalently linked graphene-porphyrins nanohybrids have been undertaken to elucidate the interactions between the two components in the ground and excited states^15^. A nanohybrid constructed from chemically converted graphene (CCG) covalently linked with porphyrins nanohybrid was prepared by a Suzuki coupling reaction of surfactant-wrapped CCG (obtained by reacting CCG with a *p*-iodobenzenediazonium salt via an aryl-addition reaction) with a porphyrin boronic ester^16^, and a graphene nanosheet covalently functionalized with cobalt[tetrakis(*o*-aminophenyl)porphyrin] (CoTAPP) was synthesized by a diazonium salt reaction, in order to improve the effectiveness of CoTAPP electrocatalysts^17^. Such organized structures are desirable because porphyrins are stable functional dyes with large extinction coefficients in the visible light region, photochemical electron-transfer ability, and rigid structures, and with potential applications in photo-harvesting and photoelectronic devices^18^,^19^,^20^. Using this approach, it is possible to design and prepare interesting functional materials combining the distinct optoelectronic properties of porphyrins and graphene.

In the current study, reduced graphene oxide was functionalized with porphyrins using the diazonium salt derivative of 5-(*p*-aminophenyl)-10,15,20-triphenylporphyrin (TPP-NH_2_); this reaction has previously been used to functionalize carbon nanotubes^21^. A stepwise approach to access the RGO-TPP nanohybrids at minimum synthetic cost was also explored. The nanohybrid materials thus prepared are stable in solution and have been characterized by a number of spectroscopic and microscopy techniques, including Fourier-transform infrared spectroscopy, Raman spectroscopy, X-ray photoelectron spectroscopy, transmission electron microscopy, and thermogravimetric analysis, which confirmed the successful purification and functionalization of the samples. Attachment of the porphyrin units to the surface of the RGO significantly improves the solubility and ease of processing of the resultant RGO-based nanohybrid materials. Ultraviolet/visible absorption and steady-state fluorescence studies indicate considerable π-π interactions and effective photo-induced electron and/or energy transfer between the porphyrin moieties and the extended π-system of RGO. The nonlinear absorption performance of these nanohybrids in the nanosecond and picosecond regimes at 532 nm has also been assessed; the nanohybrid materials exhibit increased nonlinear optical (NLO) responses in comparison with the individual RGO and porphyrin components.

## Results and Discussion

### Syntheses

**Figure 1:**
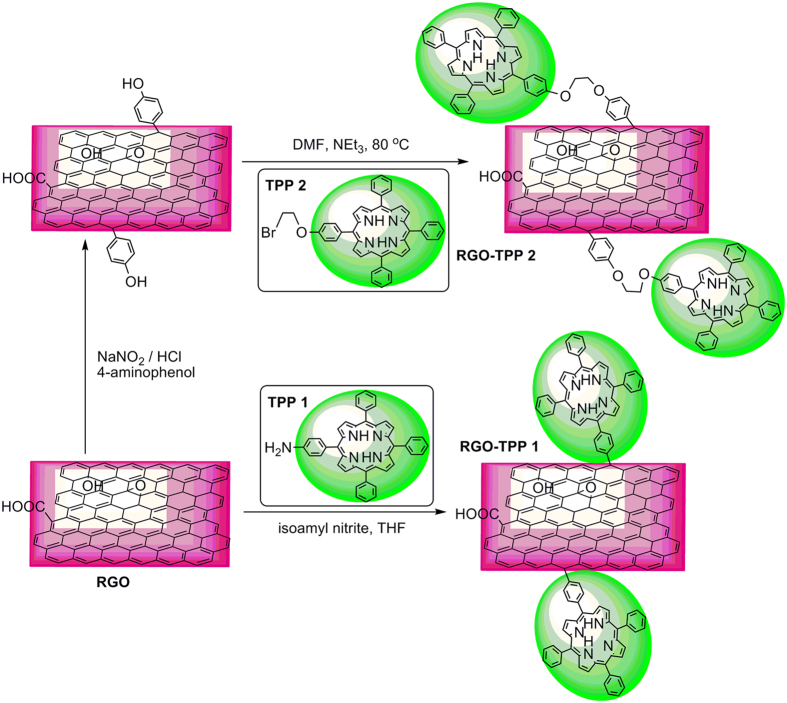
Preparation of RGO-TPP 1 and RGO-TPP 2.

**Figure 2:**
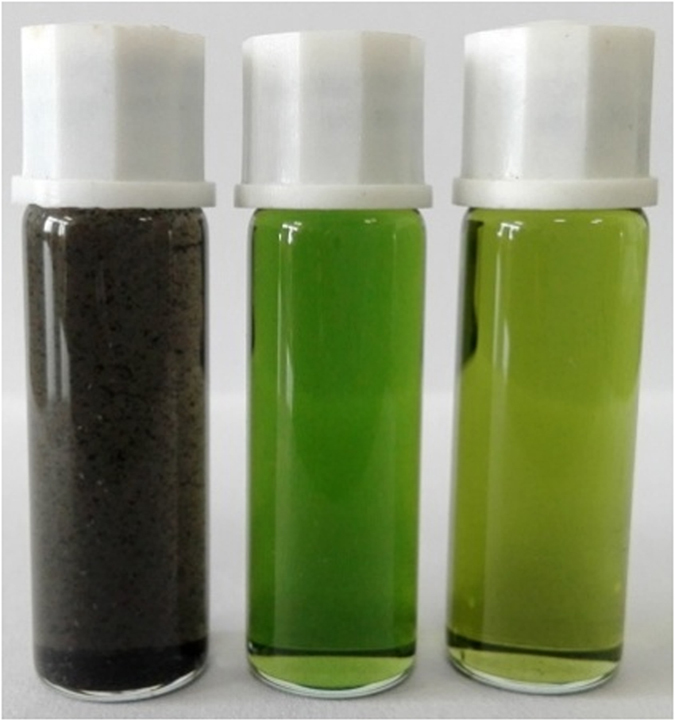
Photographic image of the samples’ dispersion in DMSO (left to right: RGO, RGO-TPP **1**, and RGO-TPP **2**).

The organic covalent functionalization of graphene can generally be divided into two categories: (a) the formation of covalent bonds between organic functional groups and the oxygen groups (including hydroxyl and carboxylic groups) of GO, and (b) the formation of covalent bonds between free radicals or dienophiles and C=C bonds of graphene^22^,^23^,^24^. Based on the previous experimental and theoretical studies^16^ the latter approach is particularly attractive due to the fact that it allows the linkage of various organic functional groups directly onto the graphene nanosheets, which may lead to a more efficient interaction between the two components and accordingly influence their photophysical and photoelectric performance. Addition of free radicals to sp^2^ carbon atoms using diazonium salt compounds is one of the most successful methods reported for the covalent functionalization of graphene^25^. Upon heating of a diazonium salt, a highly reactive free radical is produced, which attacks the sp^2^ carbon atoms of graphene forming a covalent bond^22^. Herein, we outline two different approaches for preparing porphyrin-functionalized RGO nanoplatelets. Our basic strategy involves the complete exfoliation of GO into individual GO nanosheets, followed by their *in situ* reduction with NaBH_4_ to afford individual RGO sheets. Preparing RGO sheets by reduction of GO using reductants such as NaBH_4_ is a scalable and versatile method that is well-suited to chemical functionalization, and is a method which can remove most of the oxygen-containing groups and partially restore the conductivity of the material^26^,^27^,^28^,^29^. Porphyrins were then covalently grafted onto the RGO sheets, the synthetic procedures being shown in Fig. 1. In the preparation of RGO-TPP **1**, isoamyl nitrite was used to oxidize the amino group on TPP-NH_2_ to an aryldiazo group, followed by transfer of a delocalized electron from RGO to the diazo group, forming an aryl radical after release of N_2_. The aryl radical then reacted with a carbon atom of the double bonds on the surface of RGO, changing its hybridization to sp^3^ 14,30. The porphyrin molecules were thereby covalently attached to the surface of RGO via a benzene ring. A stepwise method was also explored for the synthesis of RGO-TPP **2**. This route involves initial reaction of the aryldiazonium derivative of 4-aminophenol with RGO. Phenol units were thereby successfully immobilized on the solid surface. Subsequent grafting of TPP **2** to the surface of RGO was then easily effected via a nucleophilic substitution reaction. This stepwise method is advantageous because hydroxyl groups can be attached to the RGO surface by a single reaction without degrading the RGO electronic properties, permitting a high grafting density of organic units and potentially a wider application as an optoelectronic nanohybrid material. The as-prepared nanohybrids (RGO-TPP **1** and RGO-TPP **2**) display very good dispersibility in DMSO, with the nanohybrids’ dispersions having a light gray-green color (Fig. 2), consistent with the presence of the porphyrin units.

### Characterization

**Figure 3:**
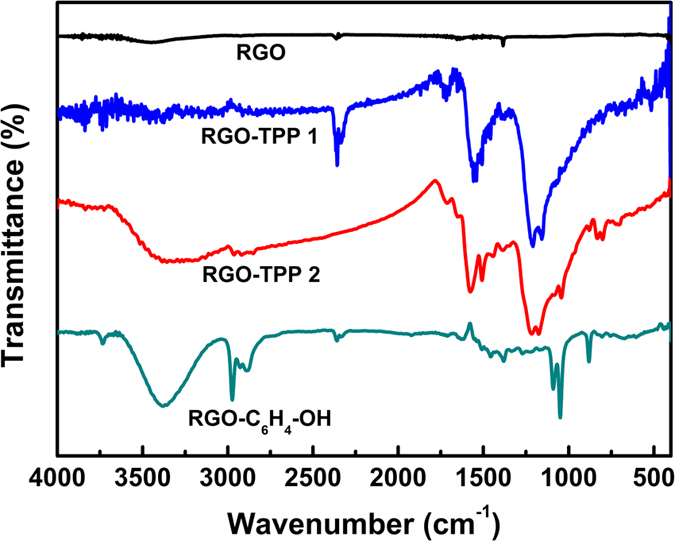
FTIR spectra of RGO, RGO-C_6_H_4_-OH, RGO-TPP 1 and RGO-TPP 2.

FTIR spectroscopy provides evidence for the reduction of the oxygen-containing groups and the functionalization of RGO by porphyrin. Figure 3 displays the FTIR spectra of pristine RGO, phenol-functionalized RGO (RGO-C_6_H_4_-OH) and the RGO-TPP nanohybrids, while for comparative purposes, the FTIR spectra of TPP **1**, TPP **2** and GO (Figures S1–S3) are also provided in the Supporting Information. As illustrated in Fig. 3, after reduction by NaBH_4_, the characteristic absorption peaks of GO nearly disappear in the FTIR spectrum of RGO. Following the diazonium treatment, new absorption bands (Fig. 3) are observed in the spectrum of RGO-TPP **1**, indicating covalent attachment to the RGO. As expected, the FTIR spectrum of RGO-C_6_H_4_-OH exhibits C-H stretching bands at 2968, 2930 and 2859 cm^−1^ that do not appear in the spectrum of pristine RGO. The absorption peak at 1023 cm^−1^ is a characteristic vibration of the *p*-disubstituted phenyl groups, and the peaks between 750-900 cm^−1^ can be ascribed to the C-H vibrations of the *p*-disubstituted phenyl groups^31^,^32^. The ν(O-H) band at ca. 3380 cm^−1^ is characteristic of phenol. After reaction with TPP **2** via nucleophilic substitution, the FTIR spectrum of the resultant RGO-TPP **2** shows some bands that are coincident with those displayed by the porphyrin unit and also observed in the spectrum of RGO-TPP **1**, confirming the covalent functionalization of RGO with porphyrin via diazotization.

**Figure 4:**
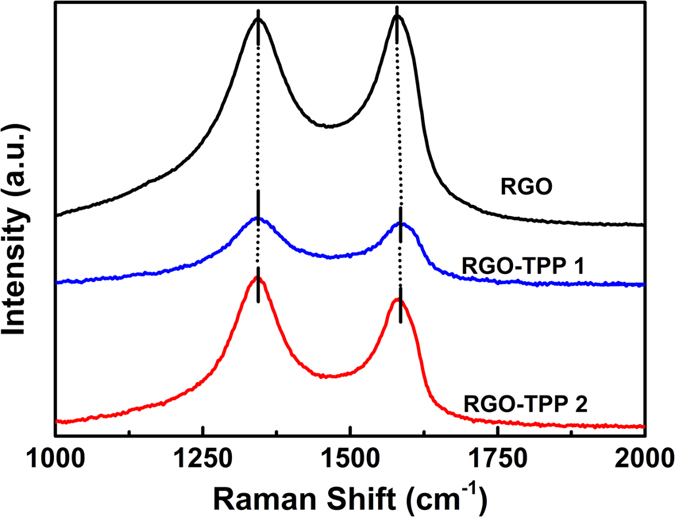
Raman spectra of RGO, RGO-TPP 1 and RGO-TPP 2.

Both reduction and covalent functionalization usually introduce defects in graphene nanosheets. The defects are often manifest in spectroscopic data, from which additional structural information, particularly the evidence for covalent functionalization, can be derived^26^,^33^. Figure 4 displays the Raman spectra of the RGO-TPP nanohybrids (RGO-TPP **1** and RGO-TPP **2**) and compares them with that of RGO. The Raman spectrum of GO exhibits a diamondoid (D) band at 1370 cm^−1^ (defects/disorder-induced mode) and a graphitic (G) band at 1602 cm^−1^ (in-plane stretching tangential mode), with an intensity ratio of diamondoid to graphitic band (I_D_/I_G_) of 0.87 (Figure S4). The D and G bands of RGO are observed at 1343 and 1577 cm^−1^, respectively, with a clear increase in I_D_/I_G_ intensity ratio (0.98) in comparison with that of GO, indicating a decrease in the average size of the sp^2^ domains upon reduction of GO and an incomplete recovery of the graphene structure^34^. After reaction with TPP-NH_2_, the D and G bands of RGO are shifted to 1340 and 1582 cm^−1^. The low-energy shift of the D-band and the high-energy shift of the G-band for RGO-TPP **1**, in comparison to RGO, are consistent with previously reported functionalization of RGO^34^. Similar results were also observed for RGO-TPP **2**, the D and G bands appearing at 1341 and 1582 cm^−1^, respectively. As compared to RGO, the I_D_/I_G_ ratios of RGO-TPP **1** and RGO-TPP **2** increase to 1.03 and 1.17, respectively, as a result of covalent functionalization, indicating that the newly formed graphitic domains are smaller in size, but more numerous in number. The intensity reactivity ratios (I_D_/I_G_)_RGO-TPP **1**_/(I_D_/I_G_)_RGO_ and (I_D_/I_G_)_RGO-TPP **2**_/(I_D_/I_G_)_RGO_ are approximately 1.05 and 1.19, respectively, which suggests a higher degree of defect formation for both nanohybrids^28^. These defects are consistent with the change of carbon atoms from sp^2^ to sp^3^ hybridization state resulting from the organic functionalization^35^. However, we have not found clear evidence of any Raman peaks characteristic of the functionalizing moieties, as a result of the low level of functionalization and/or the random orientation of the porphyrin appendages rendering the direct detection of moieties on RGO exceedingly difficult^36^.

**Figure 5:**
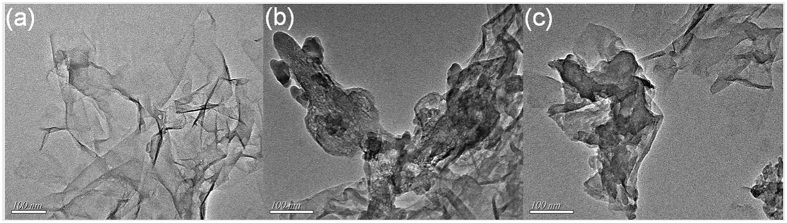
TEM micrograph of (**a**) RGO, (**b**) RGO-TPP **1** and (**c**) RGO-TPP **2**.

TEM is capable of resolving fine structure, and indeed comparison of the TEM micrographs reveals striking morphological differences. As shown in Fig. 5a, RGO displays thin wrinkled nanosheets with somewhat rough surfaces. Rather than retaining a stacked structure, the material is exfoliated into monolayer or few-layered stacks. The RGO-TPP nanohybrids display a clearly different morphology (Fig. 5b,c). The flakes appear thicker and semitransparent with a rounded morphology. The difference in morphology is consistent with covalent grafting of porphyrins onto the surface of RGO^37^. The presence of porphyrin moieties and the few-layers structure weakens the interactions between the RGO nanosheets, which improves the dispersion of RGO^38^ and was confirmed by the observations from the photographic image of the samples’ dispersion in DMSO (Fig. 2). The unique results from TEM confirm that the reaction conditions applied in this work are indeed effective in functionalizing RGO and thereby the preparation of RGO-TPP hybrid materials.

**Figure 6:**
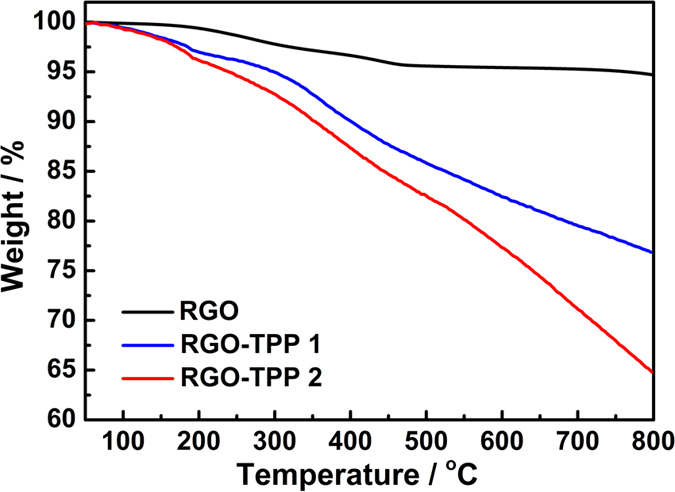
TGA curves of RGO, RGO-TPP 1 and RGO-TPP 2.

Figure 6 displays the TGA curves of RGO along with those for the nanohybrid samples RGO-TPP **1** and RGO-TPP **2** (for comparative purposes, the TGA spectra of TPP **1** and TPP **2** are provided (Figure S5 in the Supporting Information)). RGO obtained from reduction of graphene oxide is thermally stable under a N_2_ atmosphere at temperatures up to 800 ^o^C, the weight loss of ca. 5% being ascribed to the loss of adsorbed water and the remaining carboxyl, hydroxyl, or epoxy groups. After functionalization with the porphyrins, a significant increase of mass loss to 24% and 36% for RGO-TPP **1** and RGO-TPP **2**, respectively, confirms the introduction of the porphyrin moieties, but to differing degrees; the higher porphyrin content for RGO-TPP **2** is consistent with the result of UV/vis absorption, while the porphyrin incorporation is consistent with the lower decomposition temperatures found for both nanohybrids compared to RGO. The thermal stability decreases due to strong interactions leading to local distortion of the aromatic structure^39^. Distinct thermal decomposition steps cannot be clearly distinguished in the TGA curves for either nanohybrid.

**Figure 7:**
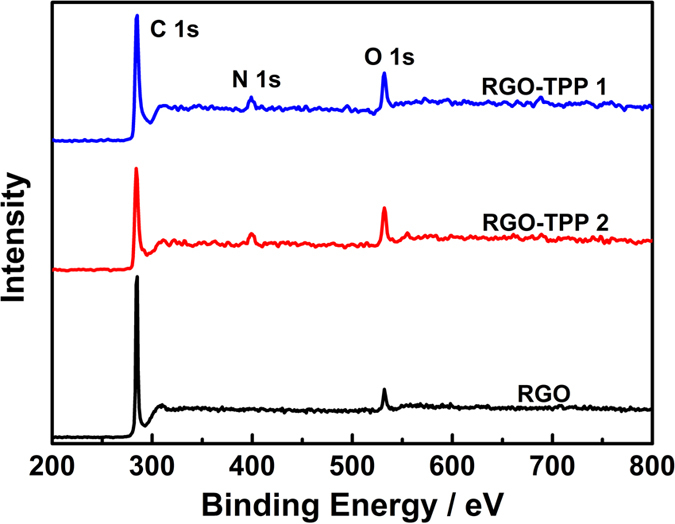
XPS wide-scan survey spectra of RGO and the chemically bonded RGO-TPP nanohybrids (RGO-TPP 1 and RGO-TPP 2).

**Figure 8:**
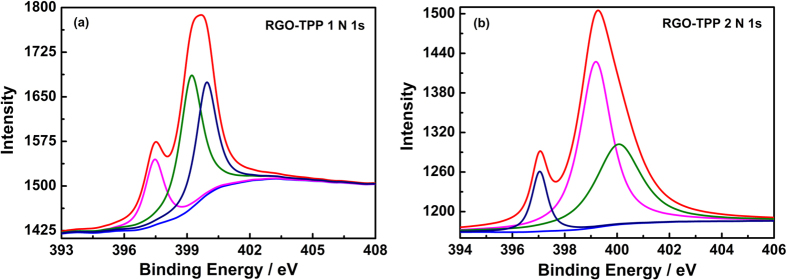
High-resolution XPS spectra of N 1 s for (**a**) RGO-TPP **1** and (**b**) RGO-TPP **2**.

Figure 7 displays the XPS survey spectra for RGO and the chemically-bonded RGO-TPP nanohybrids (RGO-TPP **1** and RGO-TPP **2**). Covalent functionalization results in the appearance of N 1 s peaks located at 399.3 and 398.4 eV for RGO-TPP **1** and RGO-TPP **2**, respectively. The C 1 s peak of RGO is observed at 284.8 eV, whereas the C 1 s peaks of RGO-TPP **1** and RGO-TPP **2** shift to 284.1 and 284.5 eV, consistent with the efficient functionalization of RGO with porphyrins. This deduction is also supported by analyzing the N region of the XPS spectra (Fig. 8). The deconvoluted N 1 s region for RGO-TPP **1** exhibits three peaks at 397.5, 399.3 and 400.0 eV, attributed to C-N, N-H and C=N^40^, respectively, confirming the covalent functionalization of RGO with porphyrins. The binding energies of the N 1 s region for RGO-TPP **2** are centered at 397.0, 399.2 and 400.1 eV and are similarly attributed. The XPS was also used to determine the weight compositions of RGO-TPP **1** and RGO-TPP **2**. Using XPS, the weight fractions of N in the samples of RGO-TPP **1** and RGO-TPP **2** were determined to be 2.40% and 2.94%, respectively, from which the porphyrin content in the nanohybrids was calculated to be 26.28% and 35.24%, respectively; the content of RGO is therefore 73.72% and 64.76% for RGO-TPP **1** and RGO-TPP **2**, respectively. Taken together, the structural data are consistent with chemical functionalization of RGO by the porphyrins.

### Photophysical properties

**Figure 9:**
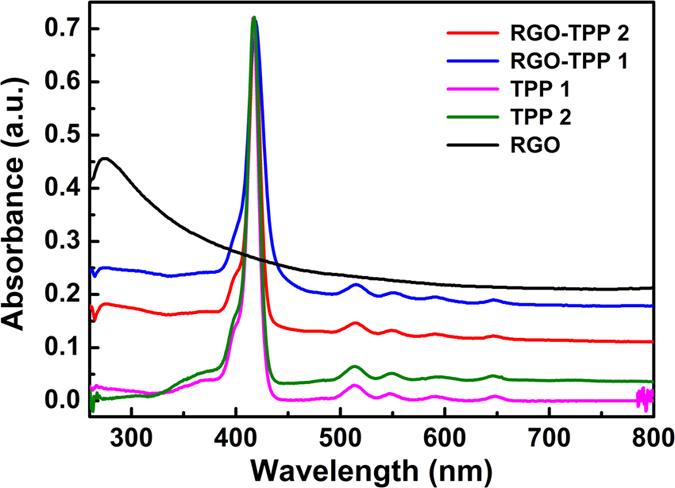
UV/vis absorption spectra of RGO, TPP 1, TPP 2, RGO-TPP 1 and RGO-TPP 2 in DMSO.

UV/vis absorption and fluorescence measurements of solutions of RGO, TPP **1**, TPP **2**, RGO-TPP **1**, and RGO-TPP **2** in DMSO were undertaken, to investigate the electronic interactions between porphyrin and RGO. For comparative purposes, the absorbance value of the Soret band of all samples was adjusted to be 0.71. As shown in Fig. 9, the UV/vis absorption spectrum of RGO exhibits a broad peak centered at 275 nm with continuously decreasing intensity extending to 800 nm, corresponding to a red shift of 42 nm compared to the corresponding peak for GO (Figure S6), and consistent with restoration of the electronic conjugation within the graphene sheets upon reduction^28^,^31^. The spectrum of the TPP **1** solution contains a strong peak at 416 nm ascribed to the Soret band of porphyrin, as well as a group of weak peaks at 513, 548, 591 and 648 nm attributed to the Q-bands of porphyrin. Two spectral changes can be observed upon covalent grafting of TPP **1** onto RGO: the Soret band diminishes in intensity and shifts to 419 nm, and the Q-bands shift to 517, 553, 593 and 647 nm. Such shifts suggest some electronic communication and the presence of strong π-π interactions between the electroactive components (porphyrin is usually regarded as the electron donor, whereas the RGO functions as an electron acceptor)^41^, but may also be partially due to the inter-graphene aggregation and/or the self-assembly of the porphyrins via π-π, hydrophobic and hydrogen-bond interactions^37^; these observations are in agreement with a previous report that the interactions of a cationic porphyrin derivative with chemically converted graphene lead to a red shift of the porphyrin Soret band^42^. Note that the absorption band of RGO in the RGO-TPP **1** hybrid material is broadened and red-shifted to 279 nm, possibly due to the interaction with porphyrin. For RGO-TPP **2**, a broad band at 418 nm is observed, corresponding to a small red shift of 2 nm in comparison with that of TPP **2**, and consistent with the introduction of porphyrins to RGO modifying the ground state spectral behavior. However, it should be noted that peak shifts observed here in UV/vis absorption spectra of the RGO-TPP nanohybrids are relatively small, especially when compared to the substantial changes routinely observed in other conjugated porphyrin constructs such as dimers, oligomers etc^43^,^44^,^45^. One reason for this may be because strong substitution effects in tetrapyrroles are typically associated with the *meso* position, whereas using the *para* position as in the current work typically has a weaker influence^43^,^45^. Upon comparing the spectra of both nanohybrids (with the absorbance of the porphyrin adjusted to be identical), we see that the overall intensity of the porphyrin-centered transitions declines on proceeding from RGO-TPP **2** to RGO-TPP **1**.This indicates that the porphyrin content in the nanohybrids increases on proceeding from RGO-TPP **1** to RGO-TPP **2**.

**Figure 10:**
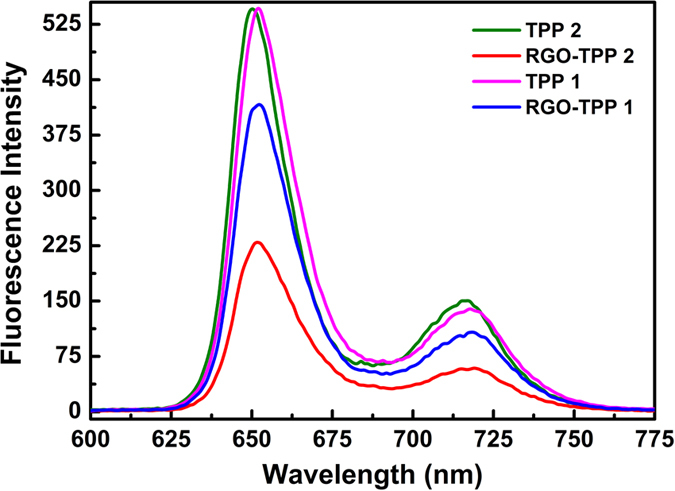
Fluorescence spectra of TPP 1, TPP 2, RGO-TPP 1 and RGO-TPP 2 in DMSO upon excitation at 419 nm, with normalization of the absorbance of the Soret band to the same value (0.71).

It is of significant interest to investigate the fluorescence behavior of materials being developed for optical nonlinearity as some NLO applications (e.g. multiphoton-excited fluorescence) necessitate high quantum yields, while other NLO studies (e.g. assessing quadratic nonlinearity by hyper-Rayleigh scattering) are complicated by fluorescence^46^. An intramolecular donor-acceptor structure usually favors the occurrence of a charge-transfer interaction or a highest occupied molecular orbital-lowest unoccupied molecular orbitals (HOMO-LUMO) electronic transition^46^. RGO can provide an ideal network to promote charge transfer in porphyrin-based systems, and transport electrons to the collecting surface. On excitation of the porphyrin moiety at 419 nm in DMSO, RGO-TPP **1** exhibits emission peaks at 651 and 718 nm (Fig. 10). The emission intensity of TPP in the RGO-TPP **1** nanohybrid is decreased by 25% compared to free TPP **1**, that is, RGO-TPP **1** undergoes fluorescence quenching, which suggests photo-induced electron and/or energy transfer from porphyrin to RGO; this is because RGO can act as a strong excimer quencher as well as a light absorber. Similar fluorescence quenching has been found for the nanohybrids of porphyrins with carbon nanotubes and fullerene; an electron and/or energy transfer mechanism has been demonstrated for these hybrid materials^40^,^47^. Following excitation at the same wavelength, the control sample (RGO/TPP **1** blends, 1:1 weight ratio with the same absorbance value of 0.71 as that of the nanohybrids) displays similar emission peaks with 8% fluorescence quenching, when compared to TPP **1** (Figure S7). The fluorescence measurements suggest that the π-π interaction between TPP **1** and RGO in RGO-TPP **1** is stronger than in the control samples, and the quenching of the porphyrin emission in RGO-TPP **1** is more effective than in the control samples. The control sample of RGO with TPP **2** exhibits similar fluorescence quenching (10%) to the physically blended sample of RGO and TPP **1** at a matching Soret band absorption (0.71). However, RGO-TPP **2** exhibits a relatively larger fluorescence quenching (58%) to that of TPP **2**. The magnitude of fluorescence quenching of RGO-TPP **2** is larger that of RGO-TP **1**, consistent with the tunability of optical properties through the different covalent functionalization processes. In addition, consistent with the results of UV/vis absorption spectra, the fluorescence emission spectra of RGO-TPP nanohybrids have practically no shift compared to free TPP, and the quantum yield has a relatively small variation as well, due to conjugation of the porphyrins in the *para* position^45^.

### Nonlinear optical properties

Pristine graphene usually settles out from organic solvents during measurements;^48^ the optical limiting properties of graphene can therefore not be studied with precision. The RGO prepared in the present work can be suspended in DMSO due to the residual oxygen-containing groups. Graphene oxide and some examples of covalently-functionalized graphene oxide (e.g. porphyrin-, phthalocyanine-, and oligothiophene-functionalized GO) have been investigated thus far^49^,^50^; however, studies on the NLO properties of RGO are still scarce^51^. Detailed investigations of the NLO response of RGO-based materials are of considerable interest, and can potentially broaden the applications of RGO-based materials in optoelectronic and photonic devices.

**Figure 11:**
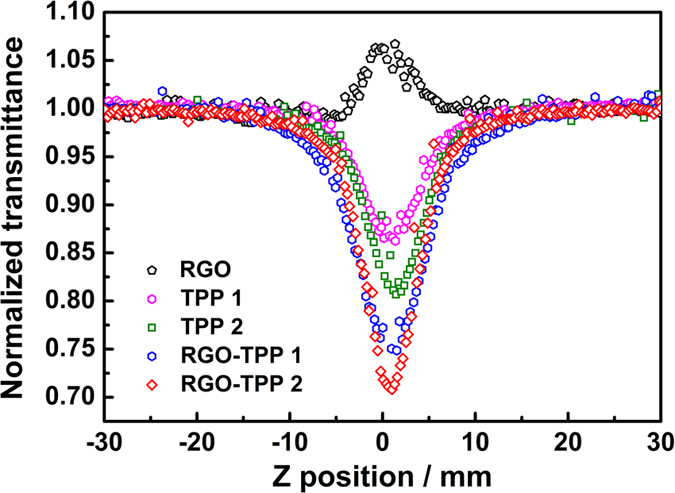
Open aperture Z-scan curves of RGO, TPP 1, TPP 2, RGO-TPP 1 and RGO-TPP 2 with 21 ps, 532 nm optical pulses.

In the present work, open-aperture Z-scan studies in both the nanosecond (ns) and picosecond (ps) regimes were undertaken to investigate the NLO performances of RGO, TPP **1**, TPP **2**, RGO-TPP **1** and RGO-TPP **2**. As a reference, a C_60_ solution was measured under the same experimental conditions. To study the nonlinear optical responses, all samples were dispersed in DMSO at a concentration of 0.15 mg/mL. Upon excitation by 21 ps 532 nm laser pulses, the saturation of absorption for RGO was clearly visible with the increase in incident light intensity, and resulting from its point band gap structure combined with Pauli blocking^52^,^53^. The Z-scan traces for TPP **1** and TPP **2** exhibit the typical valley shape corresponding to reverse saturable absorption behavior (Fig. 11), and arising from absorption from the singlet excited states^54^. Following covalent functionalization with RGO, a significant reduction in the transmission was observed for the nanohybrids RGO-TPP **1** and RGO-TPP **2**; the transmittances of TPP **1**, TPP **2**, RGO-TPP **1** and RGO-TPP **2** dropped to 85%, 80%, 74% and 70%, respectively, indicating an enhanced nonlinear absorption effect. The transmittance reductions of RGO-TPP **1** and RGO-TPP **2** are close to the decrease to 72% reported for a graphene oxide-phthalocyanine nanohybrid system with a similar excitation wavelength^51^, implying that these materials have similar NLO properties. The nonlinear absorption performances of the RGO-TPP nanohybrids are superior to the benchmark material C_60_ (Figure S8); since the shortcomings of these individual materials (porphyrins and RGO) are compensated by combining them into a single system^50^,^51^,^55^.

**Figure 12:**
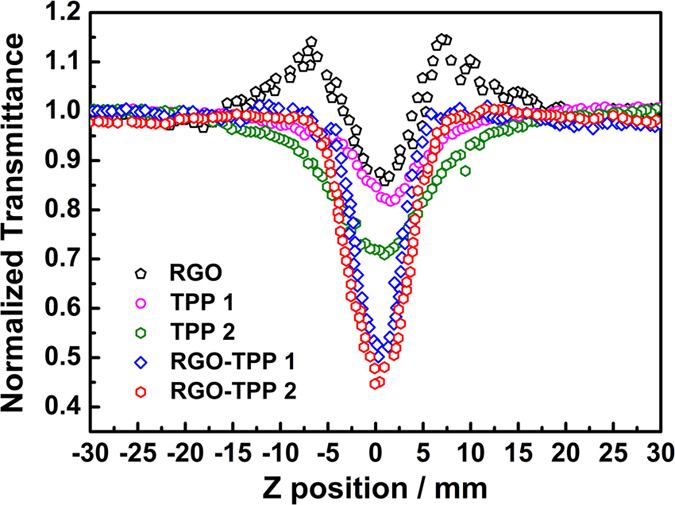
Open-aperture Z-scan curves of RGO, TPP 1, TPP 2, RGO-TPP 1 and RGO-TPP 2 with 4 ns, 532 nm optical pulses.

For comparison purposes, open-aperture Z-scan measurements were also performed at 532 nm with 4 ns laser pulses. The results clearly demonstrate that the nonlinear behavior of RGO changes from saturable absorption to reverse saturable absorption as the laser pulse length increases (Fig. 12). The transformation from saturable absorption to reverse saturable absorption indicates that another nonlinear process takes place and becomes dominant, i.e. excited-state absorption due to the large number of sp^2^-configured carbons in RGO and/or the formation of strong light scattering centers due to the vaporization of the initial particles induced by the laser pulse^55^,^56^. This interesting effect may be used for optical pulse compression, optical switching and laser pulse narrowing. Consistent with the results from employing ps laser pulses at 532 nm, TPP **1**, TPP **2**, RGO-TPP **1** and RGO-TPP **2** also exhibit decreased transmittance as they are brought close to the focal point upon excitation by 4 ns 532 nm laser pulses. Compared with the other three materials (RGO, TPP **1**, and TPP **2**), both nanohybrids show decreased transmittance at the focal point (the normalized transmittances of RGO-TPP **1** and RGO-TPP **2** are 49% and 43%, respectively), indicating a clearly improved nonlinear absorption response. In addition, and consistent with the results employing ps optical pulses, both RGO-TPP nanohybrids exhibited an improved nonlinear optical absorption response when compared to that of the standard material C_60_ (Figure S9), confirming the presence of cumulative effect due to the covalent linkage between RGO and porphyrin moieties. Optical limiting materials have drawn much attention in the past decade due to, inter alia, their potential applications in protecting delicate optical instruments from intense laser beams^57^. Carbon-based materials, e.g. fullerene^58^, carbon black^59^, graphene^60^, and carbon nanotubes^61^, all exhibit remarkabl NLO effects, including both nonlinear absorption and nonlinear scattering of intense laser beams. For an optical limiting material, the depth of the valley in the open-aperture Z-scan curves is correlated with the extent of optical limiting performance^62^. RGO-TPP **1** and RGO-TPP **2** nanohybrids should therefore have the best NLO performances in this study, with an improvement in proceeding from the ps to the ns regime due to the longer pulse width for ns excitation. The improved optical limiting response for both nanohybrids can be ascribed to a synergistic effect between two components arising from the covalent linkage. The photo-induced energy/electron transfer process between RGO and porphyrin seen in the fluorescence studies may also contribute to the nonlinear absorption. Based on these experimental results, we conclude that optical limiting properties can be improved dramatically via covalently linking RGO materials with porphyrins. Comparing the nonlinear absorption of the RGO-TPP nanohybrids with the RGO-zinc phthalocyanine nanohybrid in ref. 51, it is seen that the RGO-TPP nanohybrids have larger NLO responses, though the differing experimental geometries render such comparisons necessarily cautious. The present studies have confirmed that bonding functional materials such as porphyrins to carbon-based materials can be an advantageous approach to the pursuit of materials with enhanced solubility and NLO properties.

## Conclusions

Porphyrin-RGO nanohybrids (RGO-TPP **1** and RGO-TPP **2**) have been prepared via two synthetic routes by means of diazonium chemistry, and their linear and nonlinear optical properties have been investigated. The results of UV/Vis, Raman, TGA, FTIR, XPS and morphological studies confirm the successful fabrication of the RGO-TPP nanohybrid materials, significantly improving the solubility and dispersibility of RGO in organic solvents. Fluorescence spectra reveal quenching for the nanohybrid solutions, consistent with the presence of photo-induced electron/energy transfer between the porphyrins and RGO. Upon excitation by 4 ns and 21 ps pulses at 532 nm, RGO-TPP **1** and RGO-TPP **2** exhibit improved nonlinear optical properties compared to those of the benchmark material C_60_, and the individual RGO and porphyrins due to the strong interaction between these nanohybrid components, and suggestive of their potential as optical limiters.

## Methods

### Materials and reagents

All reactions and manipulations were carried out under a nitrogen atmosphere with the use of standard Schlenk techniques. Expandable graphite was purchased from Qingdao Zhongtian Graphite Co. Ltd. Tetrahydrofuran (THF) and other organic solvents were dried and distilled before use. Unless otherwise stated, all other reagents were purchased from commercial suppliers and used as received. Deionized water was used throughout the materials preparation process. 5,10,15,20-Tetraphenyl-porphyrin (TPP **1**), 5-(*p*-aminophenyl)-10,15,20- triphenylporphyrin (TPP-NH_2_) and 5-[4-(2-bromoethoxy)phenyl]-10,15,20-triphenyl- porphyrin (TPP **2**) were prepared according to the literature^63^,^64^.

### Instruments and measurements

Fourier transform infrared (FTIR) spectra were recorded on a MB154S-FTIR spectrometer (Canada) between 400 and 4000 cm^−1^ as KBr discs. The UV/vis absorption spectra of all samples in DMSO solution using 1 cm path-length quartz cuvettes were recorded using a JASCO V-570 spectrophotometer in the range between 200 and 800 nm. Fluorescence spectra were obtained with a Fluoro-Max-P instrument. The Raman spectra were recorded on a RenishawinVia Raman Microscope with a 532 nm excitation from a He-Ne laser. Thermogravimetric analysis (TGA) was undertaken using a Perkin-Elmer Pyris 1 system from 50 to 800 ^o^C at a rate of 10 °C/min in an aluminum crucible under a N_2_ purge. X-ray photoelectron spectroscopy (XPS) analyses were performed on a RBD upgraded PHI-5000C ESCA (Perkin-Elmer) electron spectrometer with a Mg Kα line at 280 eV. The transmission electron microscopy (TEM) experiments were conducted with a JEM-2100 (JEOL) instrument. Samples for TEM imaging were prepared by adding a drop of a dilute dispersion of the as-prepared products in ethanol onto amorphous carbon-coated copper grids and then drying in air before transfer to the TEM sample chamber.

### Nonlinear optical measurements

The open-aperture Z-scan measurements of all samples (including RGO, TPP **1**, TPP **2**, RGO-TPP **1** and RGO-TPP **2**) were carried out with linearly polarized 4 ns and 21 ps pulsed 532 nm light generated from a mode-locked Nd:YAG laser with a repetition rate of 2 Hz. In the ns Z-scan experiments, the peak intensity was 120 MW/cm^2^ for an input energy of 5.4 μJ. For the ps Z-scan measurements, the pulses had a nearly top-hat profile; the input pulse energy was 4.9 μJ and the aperture radius of the top-hat was 4.8 mm. The laser beam was focused by a 30 cm focusing lens, after spatially removing higher-order modes. To facilitate comparison, all sample concentrations were adjusted to 0.15 mg/mL; the linear transmittance of RGO, TPP **1**, TPP **2**, RGO-TPP **1** and RGO-TPP **2** was determined to be 70%, 52%, 55%, 52% and 60%, respectively. Solutions of the samples in DMSO were placed in a quartz cell of 2 mm thickness, and then moved along the axis of the incident beam (*z* direction), under computer control. The incident and transmitted laser pulses were recorded by two energy detectors (Rjp-765 energy probe), which were linked to an energy meter (Rj-7620 ENERGY RATIOMETER, Laserprobe).

### Preparation of reduced graphene oxide

Graphite powder was used to prepare exfoliated graphene oxide (GO) sheets following the modified method of Hummers and Offeman^65^. To prepare RGO, GO (200 mg) was loaded into a 150 mL three-necked flask, and DMF/water (9:1, 50 mL) was added. Ultrasonication afforded a yellow-brown dispersion. After stirring and ultrasonication for 0.5 h, NaBH_4_ (500 mg) was added and the solution was then heated in an oil bath at 80 ^o^C for 8 h. The reduced product was isolated by filtration through a 0.45 μm nylon membrane, washed with deionized water and methanol, and then dried under vacuum at room temperature for 24 h.

### Preparation of nanohybrid RGO-TPP 1

The diazotization reaction to afford the porphyrin-functionalized RGO was performed as shown in Fig. 1. In a typical experiment, RGO (40 mg) was sonicated for 0.5 h in THF (20 mL). The suspension was transferred to a 50 mL round-bottom flask equipped with a magnetic stir bar and a thermometer. The solution was deoxygenated by sparging with nitrogen for 15 min, and then TPP-NH_2_ (40 mg) and isoamyl nitrite (0.5 mL) were quickly added. The suspension was heated and stirred at reflux under N_2_ for 48 h. After the reaction was finished, the solvent was removed by evaporation, and the resultant solid residue was homogenized with dichloromethane with the aid of an ultrasonic bath. The resultant suspension was filtered through a nylon membrane, and the collected solid was washed with dichloromethane and ethanol, until the filtrate was colorless. The desired nanohybrid material RGO-TPP **1** was obtained as a black powder and was dried under vacuum overnight.

### Functionalization of RGO with TPP 2 (RGO-TPP 2)

In a typical synthesis, an aryldiazonium ion solution was prepared according to the following method. Sodium nitrite (0.117 g), 4-aminophenol (0.154 g) and sodium hydroxide (0.0398 g) were dissolved in deionized water (9 mL). The solution was then added dropwise to dilute hydrochloric acid solution (0.1 mol L^−1^, 6 mL) in an ice bath with stirring. The pH of the resultant mixture was adjusted to acidic conditions by adding further hydrochloric acid solution. The aryldiazonium ion solution was added dropwise to a previously-prepared suspension of purified RGO (16 mg) in deionized water (14 mL) with stirring. The resultant mixture was kept in the ice bath for 7 h and then at room temperature for a further 8 h. The contents were filtered through a 0.45 μm nylon membrane, and the collected solid was washed repeatedly with deionized water, ethanol and acetone to remove excess diazonium salts. This afforded the phenol-functionalized RGO hybrid as a black solid, which was vacuum-dried at room temperature for 24 h. The procedure for the preparation of RGO-TPP **2** is shown in Fig. 1. Phenol-functionalized RGO (15 mg) obtained as described above was dispersed in DMF (10 mL). Triethylamine (1 mL) and TPP **2** (15 mg) were then added and the mixture was sonicated to form a homogeneous colloidal solution. The mixture was stirred at 80 ^o^C under N_2_ for 3 days. The resulting product was isolated by filtration, and the black solid was thoroughly washed with deionized water, CH_2_Cl_2_, methanol and ethanol, and then dried under vacuum overnight. In all cases, the mass of the desired compound was greater than the mass of the starting RGO.

## Additional Information

**How to cite this article**: Wang, A. *et al*. Covalent functionalization of reduced graphene oxide with porphyrin by means of diazonium chemistry for nonlinear optical performance. *Sci. Rep.* **6**, 23325; doi: 10.1038/srep23325 (2016).

## Supplementary Material

Supplementary Information
