# Supplementary material for: Covalent functionalization of reduced graphene oxide with porphyrin by means of diazonium chemistry for nonlinear optical performance
Source: Sci Rep. 2016 Mar 24;6:23325. doi: 10.1038/srep23325 (PMC4806317; doi:10.1038/srep23325)
Supplement: Supplementary Information [file srep23325-s1.doc]

**Supporting Information**

**Covalent functionalization of reduced graphene oxide with porphyrin by means of diazonium chemistry for optical performance**

Aijian Wang^1^, Wang Yu^1^, Feng Zhou^2^, Jingbao Song^1^, Yinglin Song^2^, Lingliang Long^1^, Marie P. Cifuentes^3^, Mark G. Humphrey^3^, Long Zhang^4^, Jianda Shao^4^, Chi Zhang^1,4^,*

^1^ China-Australia Joint Research Center for Functional Molecular Materials, Scientific Research Academy, Jiangsu University, Zhenjiang 212013, P. R. China

^2^ School of Physical Science and Technology, Soochow University, Suzhou 215006, P. R. China

^3^ Research School of Chemistry, Australian National University, Canberra, ACT 2601, Australia

^4^ Key Laboratory of Materials for High-Power Laser, Shanghai Institute of Optics and Fine Mechanics, Chinese Academy of Sciences, Shanghai 201800, P. R. China

* Corresponding author, E-mail: [chizhang@ujs.edu.cn](mailto:chizhang@ujs.edu.cn), Fax: +86-511-8879-7815; Tel: +86-511-8879-7128


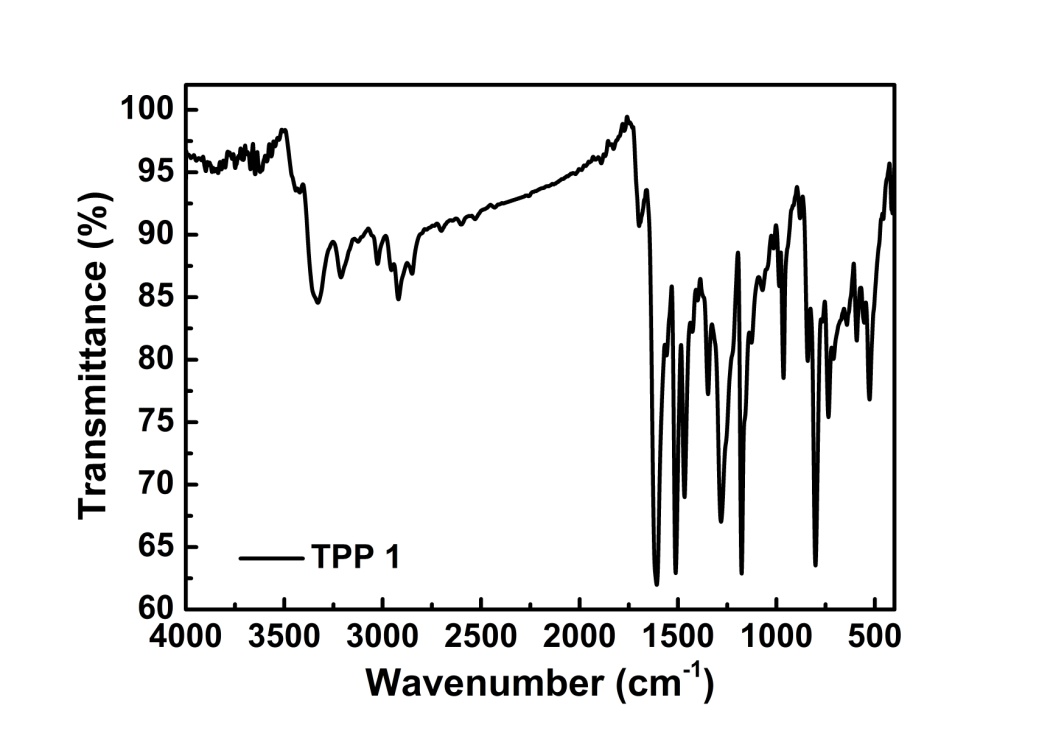


Figure S1. FTIR spectrum of TPP **1**.


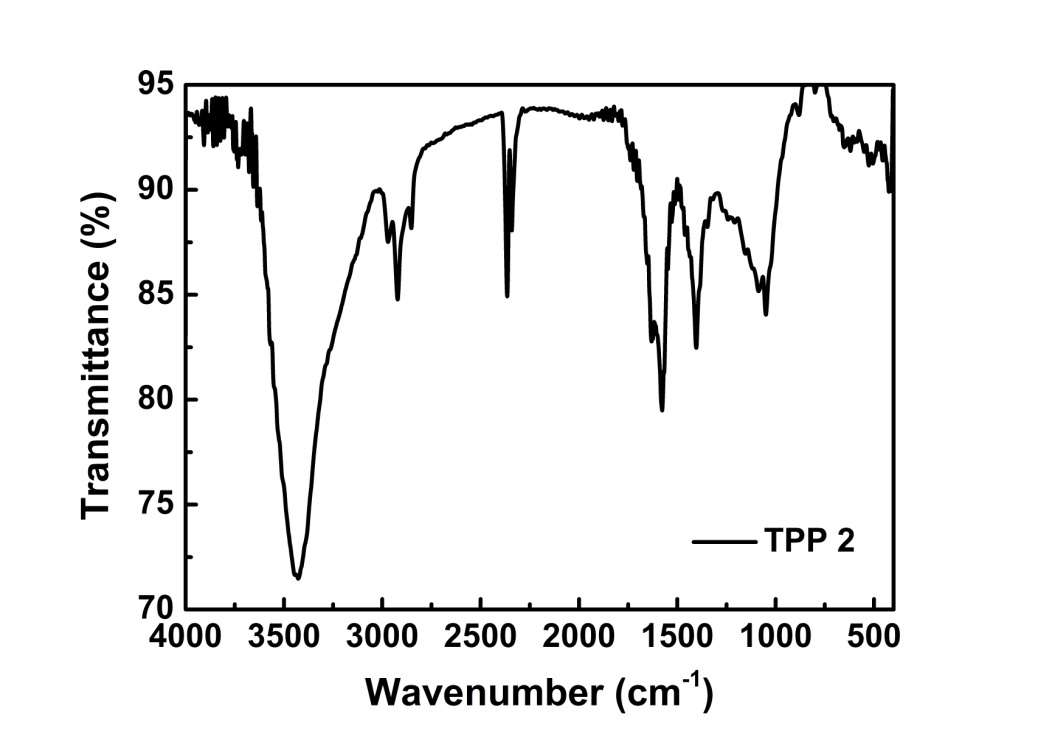


Figure S2. FTIR spectrum of TPP **2**.


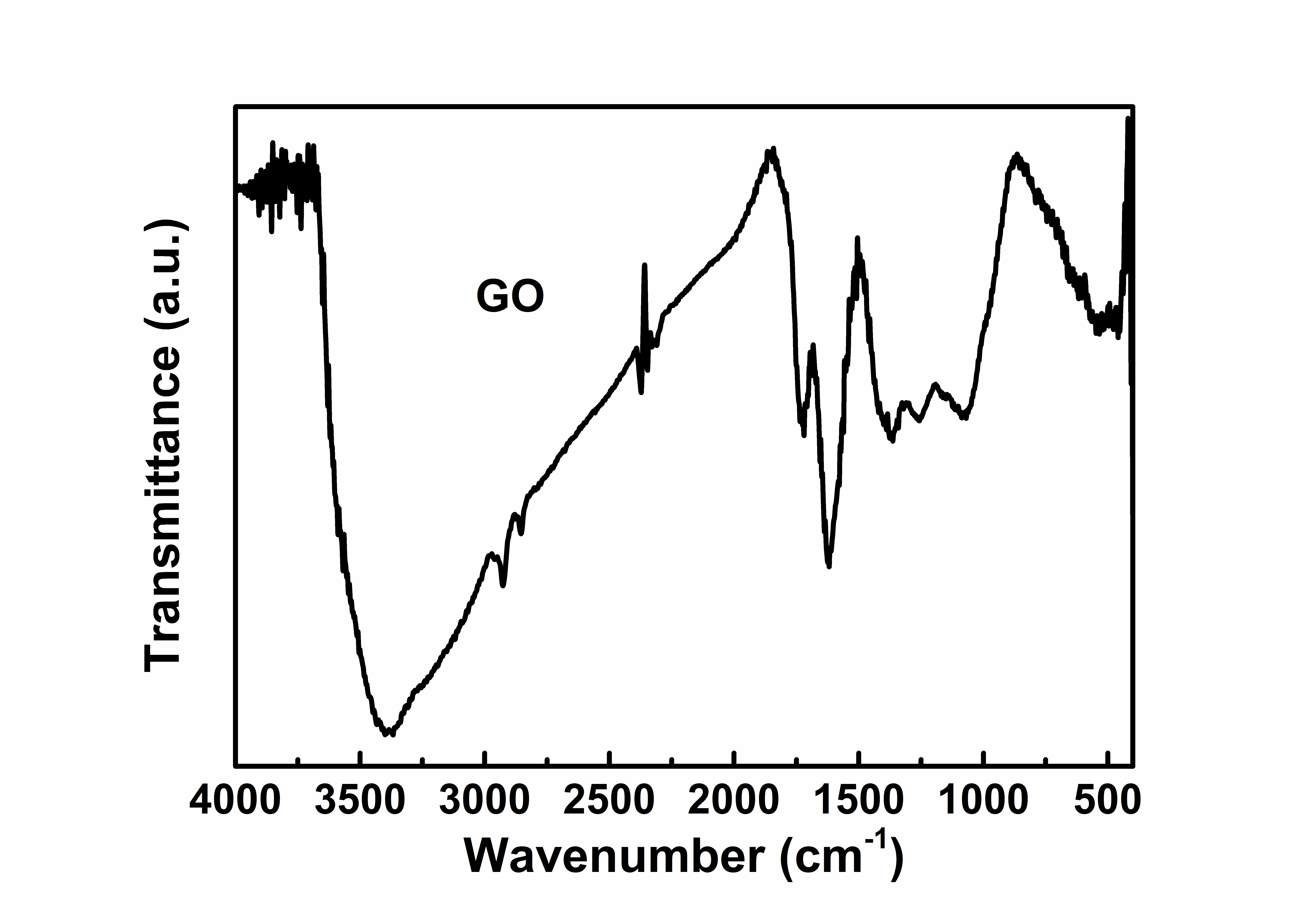


Figure S3. FTIR spectrum of GO.


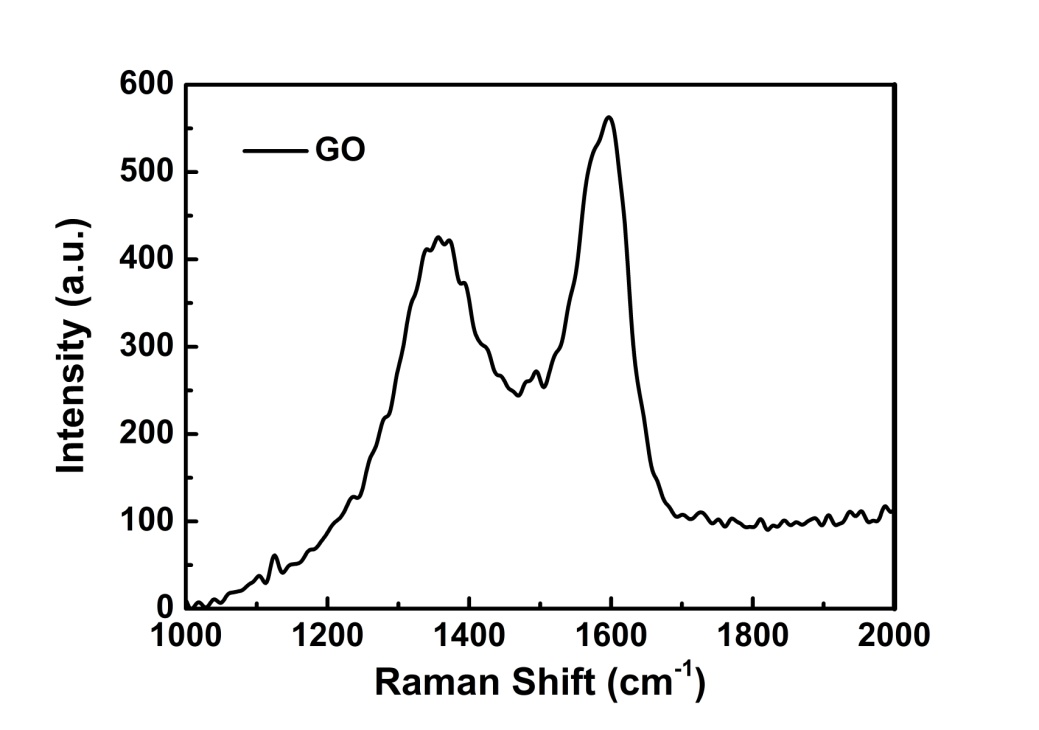


Figure S4. Raman spectrum of GO.


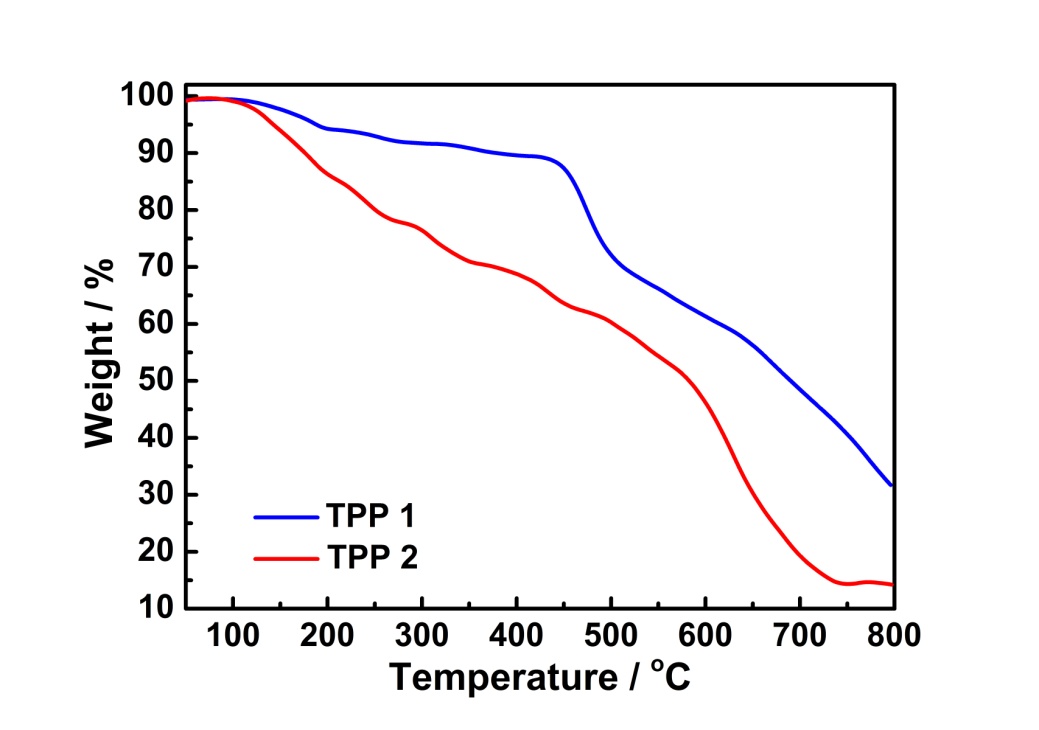


Figure S5. TGA curves of TPP **1** and TPP **2**.


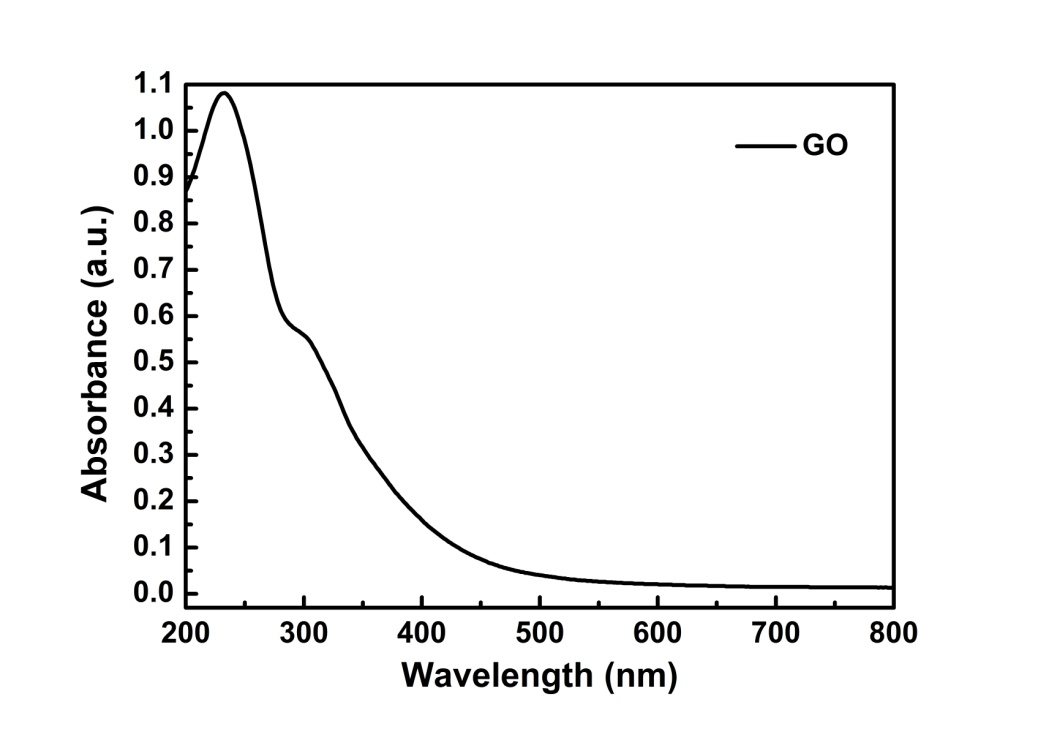


Figure S6. UV/vis absorption spectrum of GO.


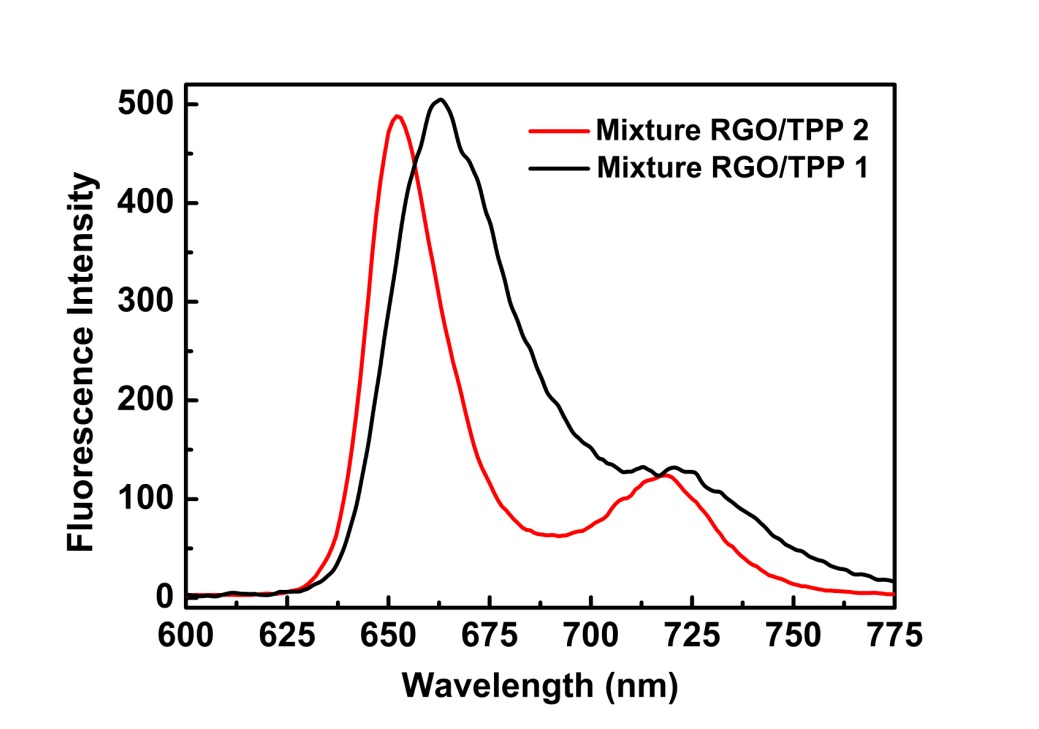


Figure S7. Fluorescence spectra of blended RGO and TPP **1**, and blended RGO and TPP **2**.


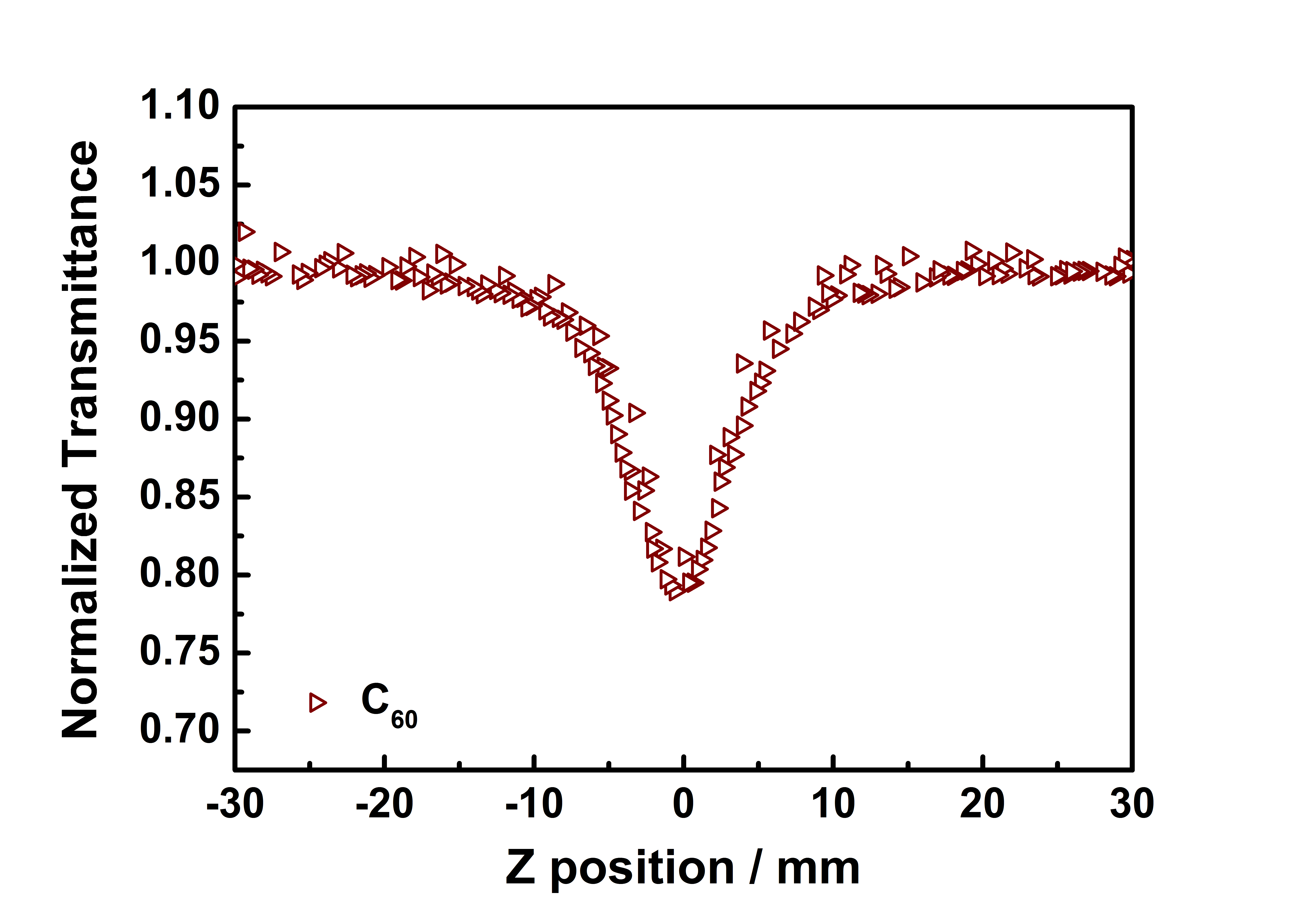


Figure S8. Open aperture Z-scan curve of C_60_ with 21 ps, 532 nm optical pulses


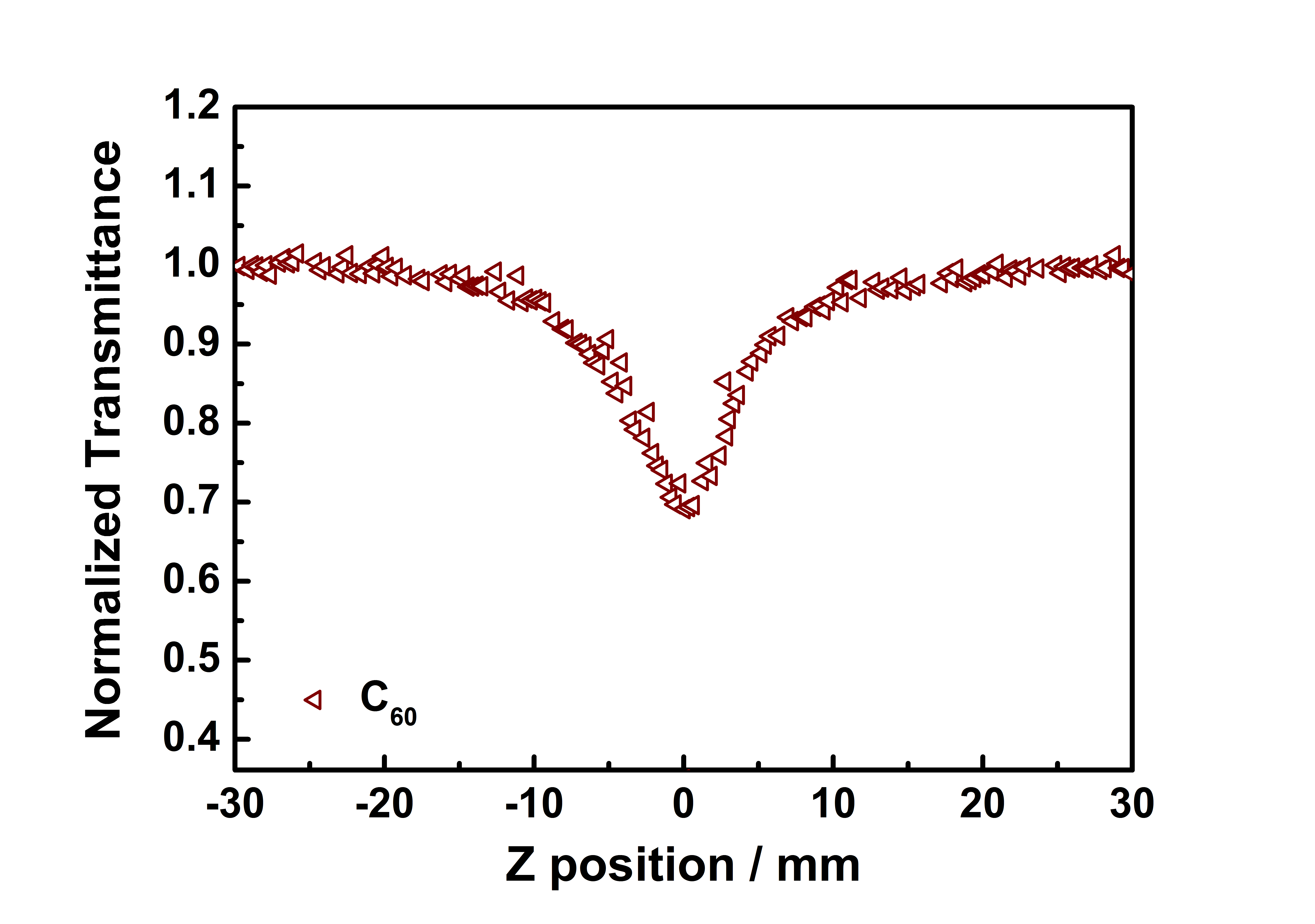


Figure S9. Open-aperture Z-scan curve of C_60_ with 4 ns, 532 nm optical pulses.
